# Strategies to Prevent Serious Fall Injuries: A Commentary on Bhasin et al. A Randomized Trial of a Multifactorial Strategy to Prevent Serious Fall Injuries. *N Engl J Med*. 2020;383(2):129–140

**DOI:** 10.20900/agmr20210002

**Published:** 2020-11-14

**Authors:** Brian C. Clark, W. David Arnold

**Affiliations:** 1 Ohio Musculoskeletal and Neurological Institute (OMNI), Ohio University, Athens, OH 45701, USA; 2 Department of Biomedical Sciences, Ohio University, Athens, OH 45701, USA; 3 Division of Geriatric Medicine, Ohio University, Athens, OH 45701, USA; 4 Department of Neurology, Ohio State University, Columbus, OH 43210, USA; 5 Department of Physical Medicine and Rehabilitation, Ohio State University, Columbus, OH 43210, USA; 6 Department of Neuroscience, Ohio State University, Columbus, OH 43210, USA; 7 Department of Physiology and Cell Biology, Ohio State University, Columbus, OH 43210, USA

**Keywords:** aging, mobility, fractures, frailty

## Abstract

Every second of every day, an older adult suffers a fall in the United States (>30 million older adults fall each year). More than 20% of these falls cause serious injury (e.g., broken bones, head injury) and result in 800,000 hospitalizations and 30,000 deaths annually. Bhasin and colleagues recently reported results from a pragmatic, cluster-randomized trial designed to evaluate the effectiveness of a multifactorial intervention to prevent fall injuries. The intervention did not result in a significantly lower rate of a first adjudicated serious fall injury among older adults at increased risk for fall injuries as compared with enhanced usual care. In this commentary we briefly review and highlight these recent findings. Additionally, we argue that the findings should not be discounted just because of the lack of statistical significance. The approximately 10% reduction compared to enhanced usual care is, arguably, meaningful at both the individual and public health level, especially when one considers that the control group had better outcomes than expected based on prior work. Moreover, we encourage future research as well as practitioners to give strong consideration to the nuances of the exercise interventions for reducing falls and fall-related injuries particularly as it relates to exercise programming specifics, namely intensity and volume, to enhance neuromuscular function and also to neurorehabilitation approaches to enhance motor function (e.g., balance, motor planning, and coordination).

## THE STAGGERING BURDEN OF FALLS

Falls are the leading cause of fatal and non-fatal injuries in older Americans [1]. Falls threaten older adults’ safety and independence and generate enormous economic and personal costs. Falls result in ~ 3 million emergency department visits, over 800,000 hospitalizations, and approximately 30,000 deaths annually [1]. The financial costs associated with fall injuries in the US is expected to be nearly $70 billion in 2020 [1]. Data from efficacy trials have indicated that exercise and multifactorial interventions are associated with fall-related benefits [2–4]. Unfortunately, however, these advances have not been successfully translated to public health practices [5]. As noted by Fixsen and colleagues, while knowing *what* to do is critical, knowing *how* to effectively implement evidence-based interventions for prevention of falls and related injuries is equally important [5]. To this end, Bhasin and colleagues conducted—and recently reported the results of—the Strategies to Reduce Injuries and Develop Confidence in Elders (STRIDE) study [6].

## SUMMARY OF THE STRIDE STUDY

The STRIDE study sought to determine the clinical effectiveness of a patient-centered intervention that combined elements of practice redesign and an evidence-based, multifactorial, individually tailored intervention for reducing fall-related injuries implemented by specially trained nurses in the primary care setting. The elegantly designed study was a pragmatic, cluster-randomized trial that included 86 primary care practices across 10 health systems. Half of the practices were randomly assigned to the intervention group and the other half were assigned to a control group (stratified by health system) with the use of covariate-constrained randomization that balanced for the size and location (rural vs urban) of the practice, as well as the race and ethnic group of the majority of persons in the practice. The participants (*n* = 2802 in the intervention group and *n* = 2649 in the control group) were community-dwelling older adults whose mean age in both groups was ~80 years. All participants were considered at increased risk for fall injuries based on each participant having one of the following: (i) a fall-related injury in prior year, (ii) two falls in the prior year, or (iii) a fear of falling because of problems with balance and walking. The rationale for including individuals with fear of falling was based on evidence indicating that fear of falling results in individuals self-limiting their activities, leading to reduced mobility and loss of physical fitness, further increasing their risk of falling [7].

The intervention group received an evidence-based, patient-centered intervention that combined elements of a multifactorial, risk factor-based, standardly-tailored fall prevention strategy that aligned with the practice guidelines offered by the Centers for Disease Control’s (CDC’s) “STEADI” toolbox and the joint American Geriatrics Society/British Geriatrics Society guidelines, and ACOVE practice change approach. Specifically, it consisted of five components, which are illustrated and described in Table 1. The control group received an information pamphlet about falls that was created by the CDC and were encouraged to discuss fall prevention with the primary care provider, who had received the results of the participant’s screening evaluation. More specifically, providers in the control group practices (i.e., usual care) received the results from the screening questions to identify age-eligible patients at high risk for falls. Additionally, a webinar about falls and fall prevention was available to the providers and staff in the control practices (based on the existing fall prevention webinar that is part of the STEADI toolkit).

The control participants received a falls informational booklet (the “Stay Independent” booklet that is part of the STEADI tool kit). This 2-page booklet included a fall risk survey and provided basic statistics on the number of falls in older adults. Additionally, it gave four fall prevention tips, which included (1) talking openly to their healthcare provider about fall risks and prevention, (2) starting an exercise program to improve leg strength and balance, (3) getting an eye exam and replacing eyeglasses as needed, and (4) making one’s home safer by removing clutter and tripping hazards. All study participants (including the controls) were told that their practices were delivering fall prevention programs. Thus, the control group was considered “enhanced usual care”.

The maximum duration of the intervention was 40-months, and the maximum duration of follow-up was 44-months. The primary outcome, assessed in a time-to-event analysis, was the first adjudicated serious fall injury. A serious fall injury was defined as a fall resulting in a fracture (thoracic and lumbar vertebral fractures excluded), joint dislocation, cut requiring closure, or hospitalization for a head injury, sprain or strain, bruising or swelling, or other serious injury. These falls were reported during telephone interviews and subsequently reviewed by an adjudication team that was unaware of treatment assignment.

The rate of first adjudicated serious fall injury did not differ significantly between the groups, as assessed in a time-to-first event analysis (events per 100 person-years of follow-up: 4.9 vs 5.3 for the intervention and control groups, respectively; hazard ratio: 0.92, *p* = 0.25). The rates of hospitalization or death were also similar in the two groups. Thus, this incredibly strong study, concluded that “the nurse-administered multifactorial intervention in a primary care setting did not result in a significantly lower rate of first adjudicated serious fall injury than enhanced usual care among older adults at increased risk for fall injuries”. The intervention did, however, result in a significantly lower rate of first *participant reported* fall injury when compared to usual care; however, the hazard ratio here was still modest (0.90).

## WHERE DO WE GO FROM HERE?

The global population of older adults (i.e., 65+ years) currently consists of ~1 billion individuals, and it is projected to increase to over 2 billion by 2050 [8]. Moreover, globally, the number of persons aged 80 years or over is projected to increase more than threefold between 2017 and 2050, rising from 137 million to 425 million [8]. Accordingly, it is clearly of dire urgency to develop pragmatic and implementable, evidence-based interventions for prevention of falls and related injuries. There are a few things we would like to note about the data from Bhasin et al. First, albeit not generally statistically significant, the intervention did exhibit a modest effect (~10% lower risk of a serious fall injury). This effect was lower than the authors originally hypothesized difference of 20%. Interestingly, the annual rates of adjudicated serious fall injuries were considerably lower in the trial than was anticipated based on findings from the LIFE Study [9] (5% vs 14%). Thus, it seems likely that the enhanced usual care control intervention exerted a modest effect that minimized the intervention vs control group differential.

On the other hand, it is possible that the intervention effect was lesser than expected based on prior individual component efficacy trials for a variety of reasons. We are of the opinion that one, the primary reason for the lack of a more robust effect likely relates to the implementation component for the “impairment of strength, gait, or balance” risk factor. Every single study participant that was assessed was determined to exhibit an “impairment in strength, gait, or balance” risk factor (i.e., 100% of the 2354 participants). Moreover, nearly all of these subjects (95.4%) prioritized and agreed to address this risk factor. This clearly suggests that this risk factor was deemed critical to address by both the specially trained nurses and the participants themselves.

The most common, and effective, therapeutic approach for enhancing strength, gait, and balance is therapeutic exercise, and a recent network meta-analysis indicates that exercise alone is the single most effective intervention for preventing injurious falls when compared to usual care (odds ratio: 0.51 (95% CI: 0.33 to 0.79)) [10]. As Bhasin et al. note, the reduced effect observed in their pragmatic trial could have been attributed to a number of reasons. With respect to exercise the trial did not (purposely) include a structured exercise intervention per se, but rather referred to community-based programs. Additionally, the adherence to the behavior modification interventions (e.g., exercise) was not monitored or reported. Thus, it is difficult to know whether the smaller than expected effects can be attributed to either of our above-mentioned suggestions of (1) low levels of sustained participation in the exercise programming, or (2) non-optimal exercise interventions being implemented; however, based on the previously reported robust effects of sustained, structured exercise interventions reducing injurious falls it seems these are two possible explanations [3,4,10]. Additionally, it is possible, if not probable, that the enhanced usual care intervention exerted a modest effect as implementation of its recommendations (e.g., exercise, vision assessment and treatment, environmental assessment and modification) have been shown to dramatically reduce fall risk [10]. It is, however, difficult to know whether these recommendations were implemented (and sustained).

The importance of habitual exercise (as well as physical activity) as it relates to healthy aging cannot be overstated. For instance, there is overwhelming evidence that lifelong exercise can delay the onset of at least 40 chronic conditions/diseases [11]. Unfortunately, only 13% of older adults meet the minimum nationally endorsed physical activity levels for aerobic exercise (at least 150 min/week) and muscle strengthening activities (at least twice/week) [12,13], and even this low number may be an overestimate (see [14] for further discussion). This has been suggested to be less likely due to a lack of knowledge about the benefits of exercise than to failures of motivation and self-regulatory mechanisms [15]. Numerous intervention programs seek to promote exercise and physical activity in later life. Unfortunately, they typically do not achieve sustained behavior change, and there has been very little increase in the exercise rate in the population over the last decade [15]. Accordingly, we advocate for further emphasis, both scientifically and clinically, on using a multipronged approach to interventions with the goal of affecting behavior change in physical activity that can influence multiple systems (physical, cognitive, and psychological health) [15].

Additionally, it should also be clearly recognized that not all exercise and/or exercise programs are created equal. For instance, it is well-known that exercise intensity, volume, and progression are keys to optimizing the exercise prescription (for further discussion see [14,16]). For instance, the effectiveness of resistance exercise training interventions for strength and muscle mass improvement is variable across studies, and a meta-analyses by Peterson et al. attempted to identify critical aspects of resistance exercise training programs that promote strength and skeletal muscle adaptation (e.g., the frequency of exercise training, the duration of exercise training, the intensity of exercise training, the volume of exercise training, etc.) [17,18]. These studies revealed two critical aspects for positive adaptations associated with progressive resistance exercise training. First, higher exercise intensity is associated with greater improvements in muscle strength (i.e., every 10% increase in contraction intensity, at least from <60 to >80% of maximum strength, resulted in a 5.3% greater increase in strength on average) [17]. Second, higher volume of resistance exercise, defined as the total number of exercise sets performed per session, is associated with greater improvements in lean mass after controlling for a selected confounds (e.g., age, study duration, gender, training intensity and frequency, etc.) [18]. Moreover, when one considers that incorrect weight shifting and trips/stumbles account for more than 60% of falls in long-term care facilities [19], it strongly suggests that exercise programming for fall prevention should not only focus on progressive resistance exercise training, but also on exercise interventions that enhance motor function. Specifically, exercises that improve motor planning, enhanced cortico-striatal control of movement (i.e., more automaticity), and increased spinal reflex gain.

Unfortunately, the common practices are not the best practices. For instance, most community-based exercise programs do not likely give consideration to therapeutic exercises that specifically challenge motor planning and control. Physical therapist would have this expertise, but for the Bhasin et al. study, it is not clear whether participants were referred to physical therapy, and if they were how long this was delivered for and at what frequency. With respect to more general exercise training many older people are unable or unwilling to embark on strenuous exercise training programs, and, despite a call from the American Physical Therapy Association [20], many seniors are often prescribed “low-dose” exercises that are physiologically inadequate to robustly increase physical function, lean mass, and strength. Unfortunately, our anecdotal observation is that there is a large degree of variability in the implementation of physical activity programs in the community-based setting, and that many of these programs lack the intensity and duration/volume to induce robust physiological and functional gains (for further discussion please see [14,16]). Thus, as Bhasin et al. point out, it is possible that the implemented community-based exercise programs may not have been evidence-based.

In summary, we commend Bhasin and colleagues for conducting an incredibly strong pragmatic trial of a multifactorial strategy to prevent serious fall injuries. In our opinion the modest effect size that was observed should not be discounted just because it did not reach statistical significance. The approximately 10% reduction compared to enhanced usual care is, arguably, meaningful at both the individual and public health level, especially when one considers that the control group had better outcomes than expected based on prior work. Moreover, we encourage future research as well as practitioners to give strong consideration to the nuances of the exercise interventions for reducing falls and fall-related injuries particularly as it relates to exercise programming specifics, namely intensity and volume, that will enhance skeletal muscle form (size) and function (e.g., strength, fatigue-resistance), but also to neurorehabilitation approaches that will enhance motor function.

## Figures and Tables

**Table 1.** Description of the five component, multifactorial fall prevention intervention.

Intervention Components	Component Details
**1. Risk Factor Assessment**	Risk factors assessed *via* questionnaire and physical exam: (1) impairment of strength, gait, or balance; (2) medications (e.g., fall risk increasing drugs); (3) orthostatic hypotension; (4) osteoporosis or vitamin D deficiency; (5) problems with feet or foot wear; (6) vision impairment; and (7) home safety.
**2. Recommendations for Risk Factor Management**	Specially trained nurses conducted patient engagement/motivational interviewing where the patients identified 1–3 recommendations to initially work on and develop action items with their respective nurse falls care manager. The care plan was approved by the participants primary care provider. When relevant, recommendations were made to the primary care provider (e.g., medication change), and/or referrals were made to health providers or community-based organizations for more detailed assessment or implementation of specific components identified in the risk assessment.
**3. Development of Individualized Care Plan**	
**4. Implementation of the Care Plan**	
**5. Follow-Up Care**	The nurse falls care manager periodically followed-up (timing based on individualized care plan) with each participant to reassess risk factors and monitor progress towards risk factor reduction. Changes in the treatment plan and alternative interventions were implemented if there was failure to improve. Note that adherence to behavior modification interventions was not routinely monitored.
